# The Antifungal Potential of Niclosamide and Structurally Related Salicylanilides

**DOI:** 10.3390/ijms25115977

**Published:** 2024-05-29

**Authors:** Bernhard Biersack

**Affiliations:** Organic Chemistry Laboratory, University Bayreuth, Universitätsstrasse 30, 95440 Bayreuth, Germany; bernhard.biersack@yahoo.com

**Keywords:** niclosamide, salicylanilides, antifungals, mycoses, neglected tropical diseases

## Abstract

Human mycoses cover a diverse field of fungal diseases from skin disorders to systemic invasive infections and pose an increasing global health problem based on ineffective treatment options, the hampered development of new efficient drugs, and the emergence of resistant fungal strains. Niclosamide is currently applied for the treatment of worm infections. Its mechanisms of action, which include the suppression of mitochondrial oxidative phosphorylation (also known as mitochondrial uncoupling), among others, has led to a repurposing of this promising anthelmintic drug for the therapy of further human diseases such as cancer, diabetes, and microbial infections. Given the urgent need to develop new drugs against fungal infections, the considerable antifungal properties of niclosamide are highlighted in this review. Its chemical and pharmacological properties relevant for drug development are also briefly mentioned, and the described mitochondria-targeting mechanisms of action add to the current arsenal of approved antifungal drugs. In addition, the activities of further salicylanilide-based niclosamide analogs against fungal pathogens, including agents applied in veterinary medicine for many years, are described and discussed for their feasibility as new antifungals for humans. Preliminary structure–activity relationships are determined and discussed. Various salicylanilide derivatives with antifungal activities showed increased oral bioavailabilities when compared with niclosamide. The simple synthesis of salicylanilide-based drugs also vouchsafes a broad and cost-effective availability for poorer patient groups. Pertinent literature is covered until 2024.

## 1. Introduction

Fungal infections pose a “silent” but constantly increasing global health problem, with more than 150 million severe cases and approximately 1.7 million deaths annually. Incidences of various mycoses are rising in line with a growing elderly and immunocompromised population in developed countries [1,2]. In addition, a considerable number of patients suffering from endemic mycoses who dearly need potent and cost-effective antifungal drugs are living in tropical and sub-tropical regions under poor health care conditions [2,3]. The emergence of drug-resistant clinical strains such as the yeasts *Candida glabrata* and *Candida auris* and the improper management of fungal neglected tropical diseases (NTDs) such as eumycetoma (also known as Madura foot) with a current treatment efficacy of only 40% are likewise alarming [4,5,6]. The exposure of vast population groups to new perilous fungal pathogens is promoted by continuing climate change and the economic exploitation of natural environments [7,8].

Currently approved antifungals belong to a confined group of molecular classes, which include 1,3-β glucan synthase inhibitory echinocandins and ergosterol-interfering azoles or polyenes [9]. Only a few derivatives from these drug classes are currently under development and/or in clinical trials, while drug candidates with other fungal targets such as HDAC (histone deacetylase, MGCD290) and DHODH (dihydroorotate dehydrogenase, olorofim) are promising but scarce, too [10,11]. Thus, new antifungal drug candidates are required to meet the growing health risk by mycoses. The repurposing of drugs applied for the therapy of other human diseases can be a treasure trove for the identification of new antifungals [12].

**Figure 1:**
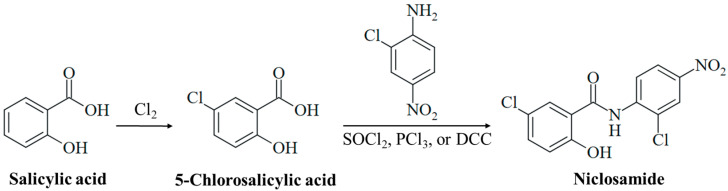
Synthesis of niclosamide. Chlorination of salicylic acid leads to 5-chlorosalicylic acid. Amide synthesis with 2-chloro-4-nitroaniline using activating reagents (e.g., SOCl_2_, PCl_3_, or DCC) generates the drug niclosamide.

In 2022, the German pharma company Bayer celebrated the 125th anniversary of the synthesis of pure acetylsalicylic acid by Felix Hofmann (1897), which was a milestone for the development of aspirin [13]. Further studies by Bayer on biologically active salicylic acid derivatives led to the discovery of niclosamide in 1953 (Figure 1), which was brought to market as a molluscicidal agent (Bayluscide/Clonitralid) against *Schistosoma*-hosting snails in 1959 and was introduced in Germany under the name Yomesan against tapeworm infections (e.g., taeniasis) in humans in 1962 [14]. Niclosamide has become an important anthelmintic drug and is currently listed as essential medicine by the World Health Organization (WHO). In the USA, the drug was approved in 1982, but Bayer withdrew it in 1996 because of economic reasons such as low profit and considerable production/availability issues in the US market [15]. Thus, it is currently not available as a human drug in the USA. In the 1950s, other salicylanilide-based bromsalans (e.g., tribromsalan) were developed as antibacterial and antifungal disinfectants, which also turned out to be molluscicidal [16,17]. The salicylanilides closantel, resorantel, oxyclozanide, clioxanide, and rafoxanide are proven anthelmintic drugs, which are applied in veterinary medicine, e.g., for the therapy of worm infections in cattle and sheep [18,19]. 

In addition to its pronounced anthelmintic properties, niclosamide also showed, inter alia, considerable activities against cancer, bacteria, viruses, diabetes, Parkinson’s disease, and rheumatoid arthritis [20,21]. The inhibition of mitochondrial oxidative phosphorylation was described as a mechanism of action of niclosamide (mainly attributed to the weakly acidic salicyl OH group functioning as a protonophor), which has been complemented by the suppression of Wnt, Notch, and STAT3 signaling over the recent years; thus, niclosamide is a pleiotropic drug with activities against a plethora of human diseases [15,21]. 

In line with the growing number of studies on the antibiotic activities of niclosamide, its antifungal potential has also garnered more interest. This review summarizes and discusses the activities of niclosamide and several notable salicylanilide analogs against various pathogenic fungi following a short summary of important chemical and pharmacological issues of this drug, which should be considered for the development of niclosamide-based antifungals. 

## 2. Methodology

PubMed, SciFinder, and Google were used for a literature search using the keywords “niclosamide”, “salicylanilides”, “oxyclozanide”, “rafoxanide”, “closantel”, “bromsalans”, and “antifungals”. In addition, PubChem was applied for additional literature search using the IUPAC names of antifungal salicylanilides as keywords. A literature survey was carried out between March and May 2024. Pertinent literature published until May 2024 was covered. 

## 3. Niclosamide: Chemistry, Pharmacology, and Antifungal Activity

### 3.1. Chemistry

A detailed understanding of the chemical constitution and properties of a drug candidate is necessary for its clinical development. Niclosamide, 5-chloro-*N*-(2-chloro-4-nitrophenyl)-2-hydroxybenzamide, is a substituted salicylanilide derivative with substituents at both aromatic rings of the molecule. The anilide ring bears a *para*-nitro and an *ortho*-chloro group. The benzoyl ring is substituted with a 2-hydroxy group characteristic of a salicyl moiety and a 5-chloro group (Figure 1). Niclosamide is lipophilic (LogP = 3.91), and its phenolic OH group is weakly acidic (pK_a_ = 6.89) [15]. It appears as a high-melting (melting point between 224 °C and 229 °C), colorless to pale yellow solid of low solubility in water (5–8 mg/L at 20 °C), which is associated with its poor bioavailability. Its solubility increases with rising pH in alkaline media, while its hydrates have shown reduced solubility when compared with anhydrous niclosamide [15]. The niclosamide salt with ethanolamine is distinctly more soluble in water (180–280 mg/L at 20 °C), and a piperazine salt of niclosamide has also been developed to improve aqueous solubility [22,23]. Of note, intramolecular H-bonding in salicylanilides can lead to two different conformations, the “open-ring” conformation (H-bond between OH and amide-NH) and the “closed-ring” conformation (H-bond between OH and amide-CO), depending on the ring substituents, which can have distinct effects on its biological activities [24].

Salicylic acid was initially obtained from the natural analgesic glucoside salicin (isolated from willow trees, *Salix* sp.) upon oxidation of the aglycone component salicylic alcohol. Technically, it is prepared by the reaction of sodium phenolate with CO_2_ under pressure (Kolbe–Schmitt synthesis) [25]. Controlled chlorination of salicylic acid leads to 5-chlorosalicylic acid (Figure 1) [26]. Niclosamide is finally synthesized from the reaction of 5-chlorosalicylic acid with 2-chloro-4-nitroaniline via activation of the benzoyl group to form the target amide (Figure 1). The activation of 5-chlorosalicylic acid is usually achieved by the formation of an acyl chloride with thionyl chloride or PCl_3_, or an *O*-acyl isourea intermediate by treatment with carbodiimides such as DCC (*N*,*N*′-dicyclohexylcarbodiimide, Figure 1) [27,28,29,30]. Thus, the synthesis of niclosamide is simple and straightforward, which is of importance in the development of a cost-effective drug against infectious diseases that mainly affect poor populations.

### 3.2. Pharmacology

**Figure 2:**
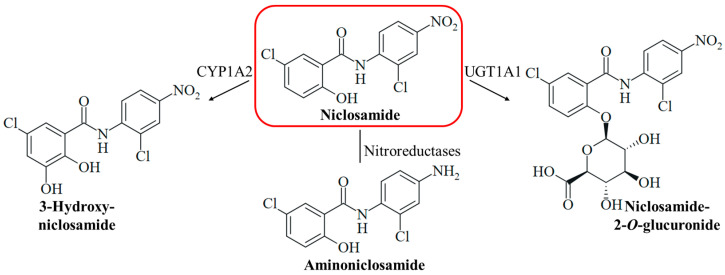
The metabolism of niclosamide can include hydroxylation catalyzed by the cytochrome P450 enzyme CYP1A2 to form 3-hydroxyniclosamide, the reduction of the nitro group by nitroreductases to form aminoniclosamide, and glucuronidation catalyzed by the UDP-glucuronosyl transferase UGT1A1 to form 2-*O*-glucuronide.

Niclosamide is a safe drug when orally administered, but injection (intraperitoneally and intravenously) of the drug can lead to adverse effects (hypopnea, sedation, and convulsions) [15]. Medicins Sans Frontières recommends a single oral dose of 2 g niclosamide for taeniasis therapy in adults and children above six years [31]. The compound has two pharmacologically problematic functional groups at the aromatic rings, a phenolic hydroxy group associated with drug metabolism such as glucuronidation (glucuronidation is a prerequisite for renal excretion), and a nitro group responsible for off-target toxicities such as genotoxicity (Figure 2). Hydrolysis of the amide bond of niclosamide is rarely occurring and considered as negligible for its pharmacology. The mutagenicity of niclosamide is higher than the mutagenic potency of its possible hydrolysis product 2-chloro-4-nitroaniline (in contrast, 5-chlorosalicylic acid itself is not mutagenic) [32]. Reduction of the nitro group by nitroreductases leads to the formation of mutagenic aminoniclosamide (Figure 2) [33]. The half-life of niclosamide is short (6.0 h), and the bioavailability is low (10%) because of its low aqueous solubility [34]. In liver microsomes, the glucuronidation of the hydroxy group to form the more soluble niclosamide-2-*O*-glucuronide is catalyzed by the UDP-glucuronosyl transferase UGT1A1, and CYP1A2-mediated hydroxylation of the activated 5-chlorosalicyl ring yields 3-hydroxyniclosamide (Figure 2) [35]. Intestinal glucuronidation is assumed to be more important for niclosamide metabolism, while hepatic glucurinidation is negligible [36]. The inhibition of glucuronidation in the liver and colon can be beneficial to increase niclosamide bioavailability; however, targeting P450 enzyme-mediated metabolism appears to be less promising [37]. CYP1A1 and Cnr nitroreductase were shown to be responsible for the bioactivation of niclosamide in *Salmonella* species [33]. Strategies to mask the hydroxy group by acylation and to remove the nitro group were applied to overcome these pharmacological drawbacks and were able to conserve the biological activities of niclosamide [38,39]. A water-soluble phosphate ester prodrug of niclosamide was also described [40]. In addition, suitable formulations of niclosamide including nano-based delivery systems were studied in order to improve its pharmacokinetics, which might be applied for the therapy of systemic mycoses as well [41,42]. The formulation as inhalation powder appears to be suitable for the specific targeting of infections of the respiratory tract [43]. Several topical formulations of niclosamide were also described and can be possible strategies to treat fungal skin infections [44,45,46]. Moreover, the grafting of niclosamide onto polymers such as hydrophilic polyurethane was described as a suitable strategy to obtain antifungal polymers [47]. However, these formulations require thorough clinical evaluation since they can also change adverse pharmacological properties of niclosamide, such as toxicities and other side effects [15].

### 3.3. Antifungal Activity

#### 3.3.1. Activity against Pathogenic Ascomycetes

**Figure 3:**
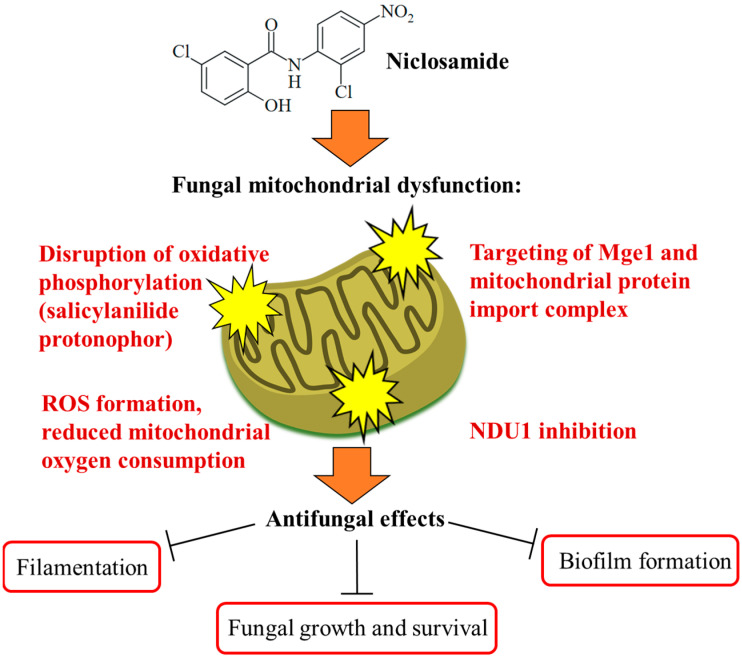
Targeting of fungal mitochondria as a crucial mechanism of niclosamide antifungal activity. Niclosamide inhibits oxidative phosphorylation and ATP synthesis. Together with the formation of toxic ROS molecules, this ultimately leads to growth arrest and fungal cell death in an efficient way. Targeting of Mge1 and the mitochondrial protein import complex was discussed as further mitochondrial mechanisms of action for niclosamide. A more specific mechanism is the inhibition of NDU1, which was accompanied by the suppression of biofilm formation.

The Ascomycota phylum is the largest fungal phylum, which harbors several pathogenic fungi [48]. Ascomycetes of the *Candida* genus are pathogenic yeasts and the most common causative agents of human mycoses [49]. *Candida albicans* is a wide-spread opportunistic *Candida* fungus, while the recently emerged multidrug-resistant *Candida auris* poses a new and considerable clinical problem requiring enhanced research efforts in terms of studies on biology, resistance mechanisms, and therapy [50]. Targeting the virulence of *Candida* species without affecting yeast growth is a promising strategy to tackle drug-resistant fungal infections without side effects. The phenotype screening of a compound library (the yeast bioactive small-molecule library, activity pre-selection in *Saccharomyces cerevisiae*) for *C. albicans* filamentation inhibition led to the identification of niclosamide as a strong filamentation inhibitor (complete inhibition at 50 µM) without growth inhibitory effects on the fungi. Niclosamide-mediated antifilamentation was also observed in azole-resistant *C. albicans* strains with overexpressed Cdr1 (*Candida* drug resistance protein 1) and MDR1 (multidrug-resistance protein 1) drug efflux pumps. In addition, niclosamide inhibited *C. albicans* biofilm production by 15% at a dose of 5 µM and reduced *C. albicans* invasion of human HT-29 colon cells as a host cell model by 20% at a dose of 40 µM. Mechanistically, a pronounced retrograde (RTG) response, which is a cell-protective mitochondria-to-nucleus signaling to compensate mitochondrial failure, was determined as a consequence of the niclosamide-mediated collapse of the mitochondrial membrane potential in treated fungi. The mitochondrial protein Mge1 was identified as a possible drug target, which is an ADP-nucleotide release factor for the mitochondrial Ssc1 heat shock protein 70 required for protein import via the Tim23-Tim17 mitochondrial protein import complex (Figure 3). It was also shown that niclosamide-mediated antifilamentation activity must be based on effects downstream of the filamentation-regulating Ras/cAMP, MAPK, and Ume6/Hgc1 pathways [51]. Another study identified the mitochondrial inner membrane protein NDU1 (NADH ubiquinone oxidoreductase complex 1) as a niclosamide target in blocking biofilm formation by NDU1-overexpressing *C. albicans* at a niclosamide dose of 10 µM (Figure 3). For in vivo studies of the anti-biofilm activity of niclosamide in a *C. albicans*-infected mouse model, Eudragit EPO nanoparticles with encapsulated niclosamide (NCL-EPO-NPs) were prepared, which conserved the anti-biofilm activity of niclosamide and increased the intracellular formation of reactive oxygen species (ROS, 32% increase at 4 µg/mL), accompanied by suppression of mitochondrial oxygen consumption (more than 90% suppression at 2 µg/mL, Figure 3). Moreover, NCL-EPO-NP (1 µg/mL) also destroyed established biofilms of fluconazole-resistant *C. albicans* and *C. auris* strains in vitro and inhibited the growth of *C. auris*. NCL-EPO-NP formulated as a gel with P407 (20%) and Poloxamer 188 (1%) was directly applicated on mucosal organs of mice, which prevented oropharyngeal candidiasis (200 µg per dose, intraorally, twice per day for four days) and vulvovaginal candidiasis (dose of 20 µg) by *C. albicans*, including fluconazole-resistant strains [52]. The putative niclosamide target NDU1 was found to be essential for *Candida* growth, virulence, and biofilm production on surfaces and media lacking glucose and plays a crucial role in Complex I of the electron transport chain in *C. albicans* mitochondria [53].

The dermatophyte *Trichophyton tonsurans* causes symptomatic and asymptomatic scalp infections and is endemic in Latin America and Africa, while it displays a rising incidence in North America with a prevalence in young Afro-American children of US cities [54,55]. Griseofulvin is applied as an antimycotic drug for the therapy of *T. tonsurans* infection, but it has shown only limited effects, which requires the development of new and more potent antifungal drugs [56]. Niclosamide was identified as a hit compound in a high-throughput screening of six compound collections for the growth inhibition of *T. tonsurans* and showed high activity against this fungus with a minimum inhibitory concentration (MIC) below 1 µM [57]. 

Chromoblastomycosis, mycetoma, and sporotrichosis are systemic fungal infections that mainly affect poor populations of (sub-)tropical regions. They were classified as NTDs by the WHO to promote and accelerate the development of drugs against these highly neglected mycoses [5]. Sporotrichosis is caused by dimorphic *Sporothrix* species and affects humans and felines, whereby the appearance of cases refractory to azole therapy as well as in vitro resistance formation raise concerns on the clinical efficacy of currently applied antimycotics in the future [58]. In particular, *S. brasiliensis* is hyper-endemic in South America and can spread to North America and Europe, necessitating the development of accurate antifungal therapies [59]. A screening of the Medicines for Malaria Venture (MMV) COVID Box for drugs with activity against a panel of 14 pathogenic fungi revealed the fungicidal activity of niclosamide (1 µM) against *Sporothrix brasiliensis*. In addition, niclosamide (1 µM) was found to be fungicidal against dimorphic *Paracoccidioides brasiliensis* and *Histoplasma capsulatum*, the causative agents of paracoccidioidomycosis and histoplasmosis [60]. A closer examination of niclosamide for the treatment of various *Sporothrix* species (*S. brasiliensis*, *S. globosa*, and *S. schenckii*, which are responsible for most sporotrichosis cases) exhibited strong antifungal activity (MIC = 0.625–2.5 µM) against 17 applied *S. brasiliensis* strains (only one further *S. brasiliensis* strain exhibited an MIC = 10 µM). Only four strains of the other *Sporothrix* species were resistant to niclosamide (MIC > 20 µM in three of four *S. schenckii* and one of four *S. globosa* strains), and a high genetic variability of *S. schenckii* was suggested as a possible mechanism of resistance. Antifungal activity was found in mycelial and yeast-like forms alike. In addition, fungicidal activity was observed in 89% of the tested *Sporothrix* strains. The broad-spectrum activity of niclosamide against *S. brasiliensis* strains endemic in Brazil and to non-wildtype strains is promising, while the resistance of most *S. schenckii* strains to niclosamide warrants more studies on the possible resistance mechanisms of this fungus [61]. 

Mycetoma (also known as Madura foot) is a chronic subcutaneous mycosis, which is subdivided into actinomycetoma caused by filamentous bacteria (mainly occurring in Mexico) and mycosis eumycetoma (prevalent in Sudan and other countries in Africa and West/South Asia). *Madurella mycetomatis* is the most common causative agent of eumycetoma [62]. Actinomycetoma is curable in most cases (curing rate > 90%) by therapy with antibiotics. However, the treatment of eumycetoma with antifungals such as itraconazole and terbinafine is strongly limited, with low curing rates between 8 and 50%, and it requires amputation at advanced stages of the disease [63]. Fosravuconazole is currently in clinical trials as a new therapy for eumycetoma, but the naphthoquinone naphthazarin has also recently revealed promising in vitro activities against *M. mycetomatis* [64,65]. A screening of a small panel of sixteen antiparasitic redox-active drugs showed high activities of niclosamide and its ethanolamine salt against two *M. mycetomatis* isolates (SO1 isolate from a Somalian patient and CBS131320 isolate of a Sudanese patient, MIC = 0.79–1.6 µg/mL). Of note, all nitroimidazole/nitrofuran- and artemisinin-based drugs tested in this study were only moderately active or inactive against *M. mycetomatis*, which indicates the outstanding role of niclosamide in targeting this pathogenic fungus. Although itraconazole was slightly more active (IC_50_ = 0.13–0.25 µM) than niclosamide, the different mechanism of action of niclosamide can add to the currently applied antifungals against eumycetoma infections [66]. The activities of niclosamide against human pathogenic ascomycetes are summarized in Table 1.

#### 3.3.2. Activity against Pathogenic Basidiomycetes

The Basidiomycota phylum forms the Dikarya subkingdom of higher fungi together with the Ascomycota phylum [67]. The human pathogenic yeast *Cryptococcus neoformans* is a basidiomycete that causes severe systemic cryptococcosis, especially in immunocompromised AIDS patients [68]. In particular, the therapy of cryptococcal meningitis with current antifungals (fluconazole and amphotericin B) is inefficient, with high mortality rates between 10 and 70% [69,70]. A screening of the LOPAC library (Library of Pharmacologically Active Compounds, Sigma-Aldrich) identified niclosamide as highly active against the *C. neoformans* H99 wildtype strain (IC_50_ = 0.17 µM) under nutrient starvation conditions mimicking host environments, and it was slightly more active than the reference drug amphotericin B (IC_50_ = 0.32 µM) against the H99 strain. Niclosamide was also active against nine *C. neoformans* clinical isolates (IC_50_ = 0.17–0.59 µM) and distinctly more active than amphotericin B against the isolates Bt27a (IC_50_ = 0.52 µM for niclosamide, 3.2 µM for amphotericin B) and NIH7 (IC_50_ = 0.48 µM for niclosamide, 2.4 µM for amphotericin B) [71]. Niclosamide showed growth inhibitory and fungicidal activities against the *C. neoformans* JEC21 isolate, with an MIC below 0.78 µg/mL and minimal fungicidal activity (MFC) of 1.56 µg/mL, while it was only growth inhibitory against the H99 strain without fungicidal effects (MIC = 1.56 µg/mL, MFC > 100 µg/mL). No growth inhibition (MIC > 100 µg/mL) was observed in *C. albicans* SC5314 or the pathogenic mold *Aspergillus fumigatus* AF293. Spore germination of *C. neoformans*, which is a crucial differentiation process of dormant spores to develop pathogenic activity and growth, was inhibited by niclosamide (germination reduction to 7.8%) at a concentration 5-fold higher than the determined MIC value [72]. Spore germination is strongly dependent on functional mitochondrial oxidative phosphorylation, and the inhibition of oxidative phosphorylation was earlier described as a suitable mechanism to suppress fungal spore germination [73,74]. Fungal spores are more resistant to stress and, thus, the strong inhibition of *C. neoformans* spore germination adds well to the antifungal portfolio of niclosamide. The activities of niclosamide against human pathogenic basidiomycetes are summarized in Table 1.

**Table 1:** Antifungal activities of niclosamide.

Phylum	Fungus	Activity/Mechanism	Reference
Ascomycetes	*Candida albicans* (including azole-resistant strains)	Inhibition of filamentation, biofilm formation, and invasion of host cells (no growth inhibition); retrograde response; possible targets Mge1 and mitochondrial protein import complex	[51]
	*Candida albicans* (including azole-resistant strains), *Candida auris*	Blocking of biofilm formation via NDU1 inhibition, increased ROS formation, suppressed mitochondrial oxygen consumption, and protection of mice from infection	[52]
	*Trichophyton tonsurans*	Strong growth inhibition (MIC < 1 µM)	[57]
	*Sporothrix brasiliensis*	Broad-spectrum growth inhibition (MIC = 0.625–2.5 µM, wildtype and non-wildtype strains); fungicidal	[60,61]
	*Paracoccidioides brasiliensis*	Fungicidal (1 µM)	[60]
	*Histoplasma capsulatum*	Fungicidal (1 µM)	[60]
	*Madurella mycetomatis*	Growth inhibition of SO1 and CBS131320 isolates (MIC = 0.79–1.6 µg/mL)	[66]
Basidiomycetes	*Cryptococcus neoformans*	Growth inhibition of H99 wildtype strain (IC_50_ = 0.17 µM) and nine clinical isolates (IC_50_ = 0.17–0.52 µM) under nutrient-starvation conditions	[71]
	*Cryptococcus neoformans*	Growth inhibitory and fungicidal effects on the JEC21 isolate (MIC < 0.78 µg/mL, MFC = 1.56 µg/mL); inhibition of spore germination	[72]

## 4. Antifungal Activity of Other Salicylanilides

### 4.1. Oxyclozanide

**Figure 4:**
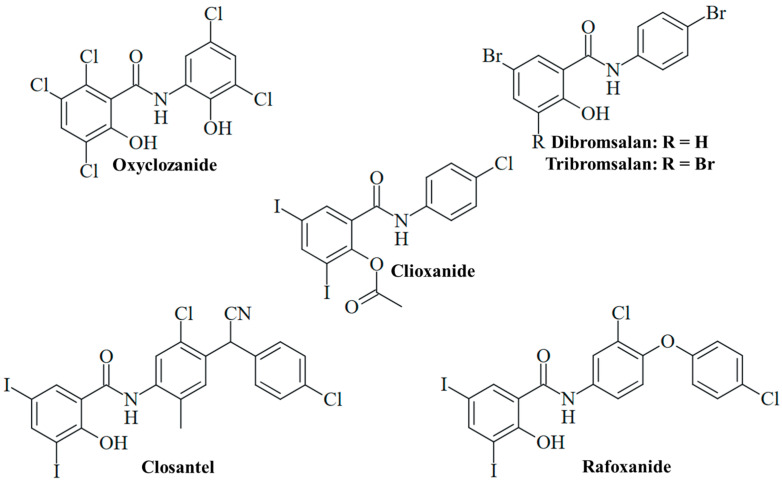
Salicylanilide-based veterinary drugs repurposed for antifungal activity.

The anthelmintic drug oxyclozanide, 2,3,5-trichloro-*N*-(3,5-dichloro-2-hydroxyphenyl)-6-hydroxybenzamide (Figure 4), has been orally applied for the treatment of adult liver flukes (fascioliasis) in cattle and sheep since the 1960s [75]. Meanwhile, oxyclozanide has also shown pronounced antifungal activities. Oxyclozanide (1 µM) was fungicidal against dimorphic *Paracoccidoides brasiliensis* and *Histoplasma capsulatum* analogously to niclosamide; however, it was inactive against *S. brasiliensis*, unlike niclosamide [60]. Oxyclozanide was also much less active than niclosamide against *M. mycetomatis* [76]. Nevertheless, oxyclozanide (20 µM) was found to inhibit *C. albicans* growth, and it also strongly suppressed filamentation (Table 2) [51]. The promising activity of oxyclozanide against *C. albicans* was studied more thoroughly thereafter. Oxyclozanide was active against azole-sensitive *C. albicans* SC5314 and 5833 isolates (MIC = 13 and 16 µg/mL, respectively) and less active against azole-sensitive S1 and 5457 isolates (MIC = 25 and 34 µg/mL, respectively), while niclosamide again showed no growth inhibitory activity at concentrations of up to 100 µM. Of great importance is the performance of oxyclozanide in drug-resistant *C. albicans* strains. It was active (MIC = 13 µg/mL) against azole-resistant HDQ-RP2 (aneuploidy Chr 5) and 6692 (overexpression of MDR1) isolates, as well as against echinocandin-resistant DPL-1007 (F641S-mutant β-1,3-glucan synthase Fks1p) and DPL-1010 (F645F-mutant Fks1p) isolates. Reduced activity was observed in the azole-resistant isolates 5674 (MIC = 25 µg/mL, overexpression of Cdr1 and Cdr2 transporters), S2 (MIC = 34 µg/mL, gain-of-function mutant Upc2 transcription factor and overexpression of Erg11 lanosterol 14-α-demethylase), and G5 (MIC = 34 µg/mL, overexpression of MDR1 and gain-of-function mutant Mrr1 transcription factor). In addition, oxyclozanide was less active against echinocandin-resistant DLP1008 (MIC = 31 µg/mL, F645P mutant Fks1p) and DLP-1009 (MIC = 25 µg/mL, F645Y-mutant Fks1p) strains. Given the established mechanism of mitochondrial oxidative phosphorylation inhibition, oxyclozanide was also tested for activity against *C. albicans* SC5314 on glucose-free medium containing non-fermentable glycerol or ethanol carbon sources, which require oxidation by the fungus via mitochondrial oxidative phosphorylation. Oxyclozanide was distinctly more active against the SC5314 strain grown on glycerol or ethanol (MIC = 5 and 3 µg/mL, respectively) than against this strain grown on glucose medium (MIC = 16 µg/mL). In addition, oxyclozanide (4 µM) disrupted the mitochondrial membrane potential in *C. albicans* cells, indicating a strong inhibitory activity against the fungal mitochondrial respiratory chain (Table 2) [77]. The described antifungal activities and MIC values of oxyclozanide are within the doses applied in sheep, which warrants further studies to develop this drug for an application as fungicide in humans [78]. Oxyclozanide displayed long half-lives in cattle (64.40 h) and sheep (21.74 h) [79]. The toxicity of oxyclozanide to rats was investigated to provide a guideline for niclosamide therapy in clinical studies with humans. Oxyclozanide revealed acute toxicity only at high oral doses (lethal dose/LD_50_ = 3707 mg/kg/day) and had no effects on blood parameters. High doses of oxyclozanide led to pathological changes of the heart, liver, and kidney [80]. Thus, oxyclozanide can be a suitable and safe drug for the therapy of infectious diseases in humans at low doses.

### 4.2. Further Salicylanilide Drugs with Antifungal Activity

#### 4.2.1. 3,5-Diiodosalicylanilides

Further anthelmintic 3,5-diiodosalicylanilides applied in veterinary medicine were studied for antifungal activities. Closantel, *N*-[5-chloro-4-[(4-chlorophenyl)-cyanomethyl]-2-methylphenyl]-2-hydroxy-3,5-diiodobenzamide (Figure 4), is an inhibitor of oxidative phosphorylation applied for the therapy of *Fasciola* and *Haemonchus* infections in domesticated animals [18]. It was suggested that inefficient worm infection therapy by closantel treatment is associated with its high activity against nematophageous fungi, which are natural enemies of nematodes. Closantel showed strong effects on *Arthrobotrys ligospora* (MIC = 0.31 µg/mL), *Duddingtonia flagrans* (MIC = 0.04 µg/mL), and *Paecilomyces lilacinus* (MIC = 0.31 µg/mL) nematophageous fungi [81]. In terms of human pathogenic fungi, closantel was identified as a hit in the screening of compound libraries for the growth inhibition of dermatophytic *T. tonsurans*, showing high activity against this fungus (MIC < 1 µM, Table 2) [57]. In sheep and cattle, oral closantel showed a long elimination half-life (2–3 weeks) and bioavailability of approx. 50%. Monoiodoclosantel metabolites were found in liver and feces [82]. Intravenous administration in sheep revealed an elimination half-life of 17 days [83]. However, considerable adverse effects of closantel were reported, such as toxicity in sheep and goats [18]. Retinal toxicity was described in humans upon ingestion of closantel pills (approx. 1500 mg), and the drug is currently contraindicated for human usage because of its severe side effects [84]. Notably, a high dose of niclosamide (oral consumption of 4 × 1250 mg) was also reported to induce retinopathy in humans [85].

Analogously to closantel, rafoxanide, *N*-[3-chloro-4-(4-chlorophenoxy)phenyl]-2-hydroxy-3,5-diiodobenzamide (Figure 4), is an anthelmintic derivative widely applied for the therapy of *Fasciola* and *Haemonchus* infections in cattle and sheep. It has a long plasma elimination half-life (7.2 days) and showed toxic effects in sheep and goats [18,83]. The treatment of lambs with oral rafoxanide was safe at doses of up to 37.5 mg/kg [86]. In cattle, the injection of rafoxanide was safe at 3 mg/kg, while eight of twelve calves treated with high doses of rafoxanide (45–60 mg/kg) developed toxicities (e.g., tremors and spasms, blindness with mydriasis and death) [87]. The drug was repurposed as an antimicrobial agent for the treatment of animal and human isolates of *C. albicans* and *A. fumigatus* with high rates of multidrug resistance, in particular, fluconazole resistance associated with upregulated Erg11 (*C. albicans*) and Cyp51A (*A. fumigatus*) resistance factors. Rafoxanide inhibited the growth of all 10 *A. fumigatus* isolates (MIC = 2–8 µg/mL) and 13 out of 15 *C. albicans* isolates (MIC = 2–8 µg/mL, Table 2). In addition, rafoxanide sensitized several fungal isolates to fluconazole treatment (and vice versa), which was correlated with the suppression of Erg11 and Cyp51A by sub-lethal doses of rafoxanide in the treated fungi (Table 2). In albino mice infected with azole-resistant *C. albicans* or *A. fumigatus*, the combination of sub-MIC doses of rafoxanide and fluconazole displayed higher protection/survival rates (70% in *C. albicans*, 60% in *A. fumigatus*, Table 2) than fluconazole monotherapy (<45% in *C. albicans*, <25% in *A. fumigatus*) [88]. Thus, rafoxanide has the potential to overcome fungal drug resistance at low doses, which warrants further investigations on its potential as an antifungal for humans. 

In contrast, the acetylated analog clioxanide, 2-(acetyloxy)-*N*-(4-chlorophenyl)-3,5-diiodobenzamide, was inactive in terms of *C. albicans* growth and lowly active regarding filamentation inhibition (Figure 4) [51]. This might be based on the protection of the phenolic hydroxy substituent with an acetyl group and the inactivation of this protonophoric substituent. Nevertheless, such *O*-acyl-modified salicylanilides can function as prodrugs and can increase oral bioavailability and plasma concentration, as in the case of the octanoyl-modified niclosamide DK-520 [38]. 

**Table 2:** Antifungal activities of veterinary salicylanilide drugs.

Compound	Fungus	Activity/Mechanism	Reference
Oxyclozanide	*Candida albicans*	Inhibition of growth and filamentation	[51]
	*Candida albicans*	Active in glucose-free medium with non-fermentable carbon sources (ethanol, glycerol); disruption of mitochondrial membrane potential	[77]
	*Madurella mycetomatis*	Low activity	[76]
Closantel	*Trichophyton tonsurans*	Active (MIC < 1 µM)	[57]
Rafoxanide	*Aspergillus fumigatus* (resistant strains with upregulated Cyp51A)	Active (MIC = 2–8 µg/mL); suppression of Cyp51A; sensitization to fluconazole; in vivo activity (sub-MIC doses) in albino mice in combination with fluconazole (survival rate 60%)	[88]
	*Candida albicans* (resistant strains with upregulated Erg11)	Active (MIC = 2–8 µg/mL); suppression of Erg11; sensitization to fluconazole; in vivo activity (sub-MIC doses) in albino mice in combination with fluconazole (survival rate 70%)	[88]
Bromsalans	*Aspergillus niger*, *Candida albicans*, *Pityrosporum ovale*	Growth inhibition; dibromsalan more active than tribromsalan against *C. albicans* and *P. ovale*; Miranol CS formulation enhanced antifungal activity	[16]
Tribromsalan	*C. albicans*	Growth inhibition (20 µM); low inhibition of *C. albicans* filamentation	[51]

#### 4.2.2. Bromsalans

The bromsalans dibromsalan, 5-bromo-*N*-(4-bromophenyl)-2-hydroxybenzamide, and tribromsalan, 3,5-dibromo-*N*-(4-bromophenyl)-2-hydroxybenzamide, are applied as disinfectants (known as temasepts) with known antibacterial and antifungal activity (Figure 4). Dibromsalan (temasept I) and tribromsalan (temasept IV) showed similar activities against *C. albicans*, while dibromsalan was more active against *Pityrosporum ovale* and *Aspergillus niger* (Table 2). The activity of dibromsalan and tribromsalan against *A. niger*, *C. albicans*, and *P. ovale* was significantly enhanced upon formulation with the cationic vehicle Miranol CS (Table 2) [16]. Tribromsalan inhibited *C. albicans* growth at a concentration of 20 µM but showed only low inhibition of *C. albicans* filamentation (Table 2) [51]. 

The absorbance of tribromsalan in rats after oral administration was approx. 65%, in contrast to only 11% of dibromsalan (comparable to 10% oral bioavailability of niclosamide). The excretion of glucuronides and sulfates of dibromsalan was described, while hydroxylated tribromsalan metabolites (4′-hydroxy-3,5-dibromosalicylanilide and probably also 5-hydroxy-3,4′-dibromosalicylanilide) were excreted as glucuronides and sulfates [89]. The considerable oral absorbance of tribromsalan is promising and warrants further antifungal studies with animals. 

### 4.3. Experimental Salicylanilide Compounds

**Figure 5:**
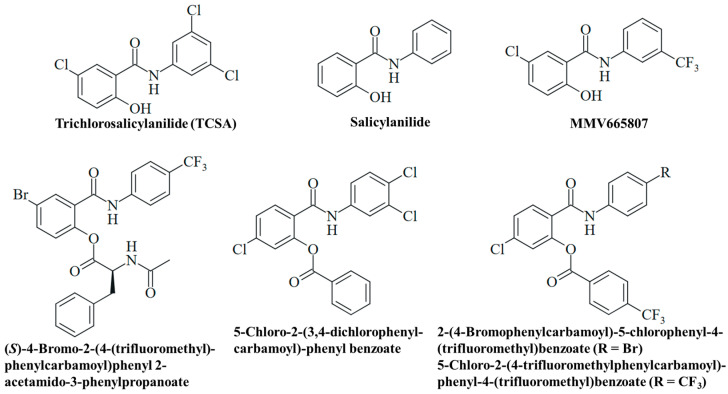
Structures of experimental salicylanilides with antifungal activities.

Experimental salicylanilides currently not in use in human and veterinary medicine were also studied for their antifungal potential. Salicylanilide and trichlorosalicylanilide (TCSA) were found to inhibit *C. albicans* filamentation (Figure 5). While salicylanilide also inhibited fungal growth analogously to oxyclozanide, TCSA solely affected *C. albicans* filamentation analogously to niclosamide both in sensitive and in azole-resistant strains (Table 3) [51]. Salicylanilide was well tolerated by mice with LD_50_ values > 500 mg/kg (intraperitoneally) and 2400 mg/kg (orally) [90,91]. Analogously to niclosamide, salicylanilide is excreted as glucuronide (in rats) [92]. Lacking the mutagenic nitro group, salicylanilide can become a valuable alternative for niclosamide in advanced antifungal studies.

The antimalarial 3-trifluoromethylsalicylanilide MMV665807 of the MMV malaria box exhibited considerable activities against the *M. mycetomatis* SO1 and CBS131320 isolates (MIC = 1.6 µg/mL for both isolates, Table 3, Figure 5). Thus, it showed roughly the same activity as niclosamide against the CBS131320 isolate and was slightly less active against the SO1 isolate [66].

Various esters of salicylanilides have revealed antifungal activities, and a selection of promising esters is provided below (Table 3, Figure 5) [93]. The *N*-acetylphenylalanine-modified compound (*S*)-4-bromo-2-(4-(trifluoromethyl)phenylcarbamoyl)phenyl 2-acetamido-3-phenylpropanoate was active against dermatophytic *Trichophyton mentagrophytes* (MIC = 3.9–7.8 µM), a fungus which contributes to the development onychomycosis (Table 3). It also exhibited considerable anti-tubercular activities (MIC = 0.25–2.0 µM), including multidrug-resistant strains as well as high selectivities for the microbes (selectivity index = 24.1–194.8), indicating a reduced general toxicity of this compound [94]. In addition, 5-chloro-2-(3,4-dichlorophenylcarbamoyl)phenyl benzoate has shown distinct activity against *Trichosporon asahii* (MIC = 3.9 µM), the causative agent of trichosporonosis, and moderate activity against *Candida krusei* (MIC = 15.6 µM) and *T. mentagrophytes* (MIC = 31.3 µM), while it was inactive against *C. albicans* (Table 3). A change in the medium to slightly acidic conditions (approx. pH 5) led to marginally reduced activities for this benzoate compound [95]. In addition, benzoates from 4-trifluoromethylbenzoic acid were studied. As expected, the compounds 2-(4-bromophenylcarbamoyl)-5-chlorophenyl-4-(trifluoromethyl)benzoate (ClogP = 6.74) and 5-chloro-2-(4-(trifluoromethyl)phenylcarbamoyl)phenyl-4-(trifluoromethyl)benzoate (ClogP = 6.59) revealed greater lipophilicity (based on the benzoyl groups) than their salicylanilide precursors. But they also showed higher activity against *T. mentagrophytes* (MIC = 0.49 µM) than their phenolic parent compounds (Table 3). However, some compounds of this series of 4-trifluoromethylbenzoates were insoluble and could not be tested for antifungal activity, in contrast to the corresponding salicylanilides [96]. Further examples of promising antifungal *O*-acyl salicylanilides include pyrazine-2-carboxylates and carbamates with distinct activities against *T. mentagrophytes* and molds [97,98]. 

**Table 3:** Antifungal activities of experimental salicylanilide drugs.

Compound	Fungus	Activity/Mechanism	Reference
Salicylanilide	*Candida albicans*	Inhibition of growth and filamentation	[51]
TCSA	*Candida albicans* (including azole-resistant strains)	Inhibition of filamentation; no growth inhibition	[51]
MMV665907	*Madurella mycetomatis*	Active (MIC = 1.6 µg/mL, SO1 and CBS131320)	[66]
(*S*)-4-Bromo-2-(4-(trifluoromethyl)phenylcarbamoyl)phenyl 2-acetamido-3-phenylpropanoate	*Trichophyton mentagrophytes*	Active (MIC = 3.9–7.8 µM)	[94]
5-Chloro-2-(3,4-dichlorophenylcarbamoyl)phenyl benzoate	*Trichosporon asahii*	Active (MIC = 3.9 µM); medium acidification (pH 5); reduced activity	[95]
	*Candida krusei Trichophyton mentagrophytes*	Moderate activity (MIC = 15.6 µM, *C. krusei*; MIC = 31.3 µM, *T. mentagrophytes*)	[95]
2-(4-Bromophenylcarbamoyl)-5-chlorophenyl-4-(trifluoromethyl)benzoate 5-Chloro-2-(4-(trifluoromethyl)phenylcarbamoyl)phenyl-4-(trifluoromethyl)benzoate	*Trichophyton mentagrophytes*	Active (MIC = 0.49 µM)	[96]

### 4.4. Structure–Activity Relationships

**Figure 6:**
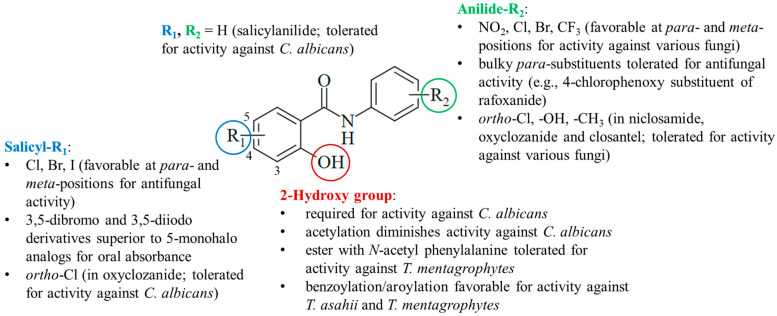
Structure–activity relationships of antifungal salicylanilides.

The current knowledge of antifungal salicylanilides allows the determination of preliminary structure–activity relationships (Figure 6). Preferentially, the salicyl moiety has weakly electron-withdrawing halogen substituents (Cl, Br, and I) at the *para*- and *meta*-positions, in particular, at position 5. Compounds with strong electron-withdrawing nitro substituents were largely absent in antifungal studies probably because of their distinctly lower antimicrobial activities published before, whereas weaker electron-withdrawing halogens (Cl and Br) were also beneficial for anti-tubercular activity [99,100]. 3,5-Dibromo- and diiodosalicylanilides often possessed increased oral absorbance and prolonged elimination times when compared with 5-monohalo analogs. However, it is difficult to evaluate the beneficial effects of substituents since salicylanilide lacking any substituent in addition to the 2-hydroxy group also possessed considerable activity against *C. albicans*. Together with the low activity of the *O*-acetyl derivative clioxanide, this highlights the importance of the salicyl hydroxy group for activity against *C. albicans*. For the treatment of other pathogenic fungi, esterification of the 2-hydroxy with various substituted benzoyl and aroyl derivatives as well as *N*-acetyl amino acids was tolerated or even improved antifungal activity when compared with their salicylanilide precursors. 

The anilide ring substituents of active salicylanilides are diverse in nature, number, and position. Electron-withdrawing *meta*- and *para*-substituents (NO_2_, Cl, Br, and CF_3_) are favorable for antifungal activity. This is in line with the structure–activity relationships of anti-tubercular salicylanilides [99,100]. Fluorine substituents were less tolerated in tests with *Candida albicans* [51]. Although the absence of the *para*-nitro group retained the inhibition of oxidative phosphorylation, a more recent study disclosed a distinctly reduced activity of the niclosamide analog lacking the *para*-nitro substituent [39,101]. Bulky *para*-substituents were also tolerated in active salicylanilides such as rafoxanide. In addition to niclosamide, *ortho*-substituents are found in active salicylanilides such as oxyclozanide.

## 5. Discussion of Current Challenges and Opportunities

### 5.1. Antifungal Activity and Mechanisms

The promising effects of niclosamide on various cancers and infectious diseases have shed a new light on this simple salicylanilide-based drug and enhanced the efforts to develop it as a salient drug for the treatment of systemic human diseases such as mycoses. The described antifungal properties of niclosamide and related salicylanilides are sound and versatile. Fungicidal activity as well as the inhibition of fungal growth, filamentation, and spore germination were observed in addition to the well-established niclosamide-mediated interference with oxidative phosphorylation and mitochondria damage. Niclosamide shares its activity as a protonophor with other salicylanilides (due to the slightly acidic OH group) and inhibits oxidative phosphorylation and ATP synthesis. Together with the formation of toxic ROS molecules, this ultimately leads to growth arrest and fungal cell death in an efficient way [52]. Further mitochondrial targets include Mge1 and the mitochondrial protein import complex, as well as the NDU1 protein associated with biofilm formation [51,52]. 

The antifungal properties of salicylanilides can also be based on further mechanisms, such as interference with vital signaling pathways as determined for niclosamide in cancer cells [23]. A very promising downstream effect of the salicylanilide rafoxanide is the suppression of eminent fungal drug targets such as Erg11 and Cyp51A, which is especially auspicious in terms of combination therapies with already approved antifungals [88]. The in vivo synergy effects of the uncoupling agent rafoxanide with the azole drug fluconazole are remarkable since mitochondrial perturbation and the uncoupling of oxidative phosphorylation were reported to be responsible for the upregulation of efflux pump expression associated with resistance to azoles and other antifungals [88,102,103,104]. This can have an impact on the development of the promising Gwt1 inhibitor manogepix, which turned out to be less active against fungi upon the upregulation of efflux pumps too [105]. Moreover, there is evidence that the activity of MDR-associated efflux pumps depends on the pH value of the cellular environment, which might be a possible target for protonophors such as salicylanilides [106]. Growing knowledge of the antifungal immune response can also be applied for an improved therapy of human mycoses [107]. Of note, niclosamide has shown eminent immunomodulating activities and successfully passed a clinical pilot study for the treatment of rheumatoid arthritis, leading to improved quality of life [108]. 

The antifungal activity of salicylanilides affects a broad spectrum of pathogenic fungi of the Ascomycota and Basidiomycota phylae, including multidrug-resistant strains and clinical isolates. Oxyclozanide was active against azole-resistant and echinocandin-resistant *C. albicans* isolates, for example, in [77]. A detailed understanding of the pharmacokinetic drawbacks of niclosamide can contribute to the development of niclosamide as a new potent antimycotic drug. Chemical modifications and sophisticated formulation systems can solve these problems. It is noteworthy that salicylanilide analogs without the genotoxic nitro group also exhibited antifungal activities. A chemical fine-tuning of the salicylanilide structure can improve antifungal activity, selectivity for the pathogen, and pharmacological properties. For instance, the substitution pattern of salicylanilides was decisive for *C. albicans* growth and filamentation inhibition, while the modification of the phenolic hydroxyl group by benzoylation has led to considerable activity against *T. mentagrophytes* [51,95,96]. Such acylated salicylanilides have the potential to prevent glucuronidation and associated renal elimination. The niclosamide scaffold was meanwhile also successfully replaced by Schiff base and acyl hydrazone bridged antifungals bearing halogenated salicyl moieties [109,110]. The structurally even simpler 2-chloro-*N*-phenylacetamide has likewise revealed promising activities against *Aspergillus* and *Candida* species, including drug-resistant strains, while it exhibited promising DHFR (dihydrofolate reductase) inhibitory and ergosterol-targeting mechanisms of action [111,112,113,114]. How far these promising modified compounds will have an impact on the development of new antifungals remains to be shown. The breach of drug resistance and the sensitization of fungi to currently applied antimycotics are definitely criteria for antifungal drug candidates with the potential to reach advanced stages of clinical development.

### 5.2. Salicylanilide Resistance and Possible Combination Partners

While niclosamide and some salicylanilide derivatives were especially active against drug-resistant fungal strains, it cannot be ruled out that a prolonged therapy of mycoses can develop niclosamide resistance. Acquired niclosamide resistance in bacteria was associated with nitroreduction by enhanced nitroreductase activity. This finding provides a strategy to circumvent resistance by other drugs that are activated via nitroreductase [115]. In patients suffering from niclosamide-resistant beef tapeworms (*Taenia saginata*), treatment with the nitro-thiazole drug nitazoxanide was able to overcome niclosamide resistance [116]. Notably, nitroreductases also play a vital role in fungi [117]. ROS formation and oxidative stress by the naphthoquinone menadione were increased by fungal nitroreductase in *Aspergillus nidulans* [118]. However, it remains to be elucidated how far fungal nitroreductases contribute to niclosamide activity and resistance, in particular since salicylanilides lacking the nitro group also showed pronounced antifungal activities. Intrinsic resistance in fungi might be attributed to the overexpression of transporters (Cdr1 and Cdr2) and Erg11 lanosterol 14-α-demethylase, the mutation of transcription factors (Mrr1), and certain Fksp1 mutants, as observed from a study with oxyclozanide using resistant *C. albicans* isolates [77]. Drugs tackling these factors can be suitable combination partners for salicylanilides. In addition, the high genetic variability of niclosamide-resistant *S. schenckii* was suggested as a possible resistance factor, in contrast to *S. brasiliensis* strains that are highly sensitive to niclosamide treatment [61]. In leukemia cells, a CRISPR/Cas9 library screen in the presence or absence of niclosamide identified DHODH and the heat-shock protein HspA9 (also known as mitochondrial Hsp70 and mortalin) to be overexpressed in surviving cells, and inhibitors of these targets are expected to generate synergy effects with niclosamide [119]. Olorofim is a reversible DHODH inhibitor with high bioavailability (82%) and a long half-life (30 h) that has reached advanced clinical trials for invasive fungal (mold) infections [120,121]. Since it is the first member of a new class of antifungals, olorofim adds well to the current arsenal of antifungal drugs. As a potent DHODH inhibitor, it might be a suitable combination partner for niclosamide and its analogs in future antifungal studies. Experiments with cancers showed enhanced activities of antimetabolites such as cytarabine (ara-C) and gemcitabine in cells treated with niclosamide [122,123]. Flucytosine is an approved antifungal antimetabolite, and, therefore, it might be considered for the combination with niclosamide in antifungal assays as well [124]. Several HspA9/mortalin inhibitors (e.g., mortaparibs and cationic rhodacyanines) with anticancer activities were described that might be tested for antifungal activity in combination with niclosamide [125]. Of note, Hsp90 inhibitors are currently under investigation as possible antifungal agents [126]. These drug combinations are also expected to be promising treatment options for infections with azole-resistant fungi.

### 5.3. Adverse Effects, Drug–Drug Interactions, and Drug Availability

Severe visual disorders and retinopathies were described in humans who have consumed higher (toxic) doses of salicylanilide drugs such as niclosamide or closantel [84,85]. These visual adverse effects can be reversible and were treated successfully with corticosteroids [127]. However, the risk to induce retinopathy by a new antifungal drug should be minimal and needs to be considered for the development of niclosamide and salicylanilide antimycotics in the future. Acute toxicities at high doses may also include negative effects on vital organs such as the heart, liver, and kidneys [80]. Since low doses of niclosamide and the salicylanilides oxyclozanide and rafoxanide already exhibit pronounced antifungal activity in vitro and/or in vivo, the application of non-toxic but antifungal doses in humans appears to be possible. The influence of niclosamide on the efficacy and toxicity of other drugs should also be taken into account. The described inhibition of CYP1A2 and CYP2C8 by niclosamide can have distinct effects on other drugs applied for the therapy of viral infections (e.g., CYP1A2-mediated metabolism of clopidogrel, clozapine, and theophylline and CYP2C8-mediated metabolism of ibuprofen, loperamide, morphine, pioglitazone, remdesivir, repaglinide, and rosiglitazone) [36]. Niclosamide was also shown to inhibit two organic anion transporters (OAT1 and OAT3) and the organic cation transporter 2 (OCT2), leading to a reduced renal elimination of the drugs furosemide and metformin in treated rats [128]. These drug–drug interactions might be potentiated in combination with other antifungals such as azoles, which can also target renal transport protein systems including OATs, OCTs, and P-gp [129]. Such interactions are not necessarily negative for the patient. The antidiabetic drug metformin, for example, also showed antifungal properties and even enforced the activity of azoles and amphotericin B against *C. glabrata* [130]. In addition, synergy effects were described for the combination of niclosamide with metformin in colorectal cancer [131]. Thus, a detailed understanding of the antifungal and pharmacological properties of salicylanilides can pave the way for improved therapies of mycoses in the future.

Despite the described toxicities at high doses and possible drug–drug interactions that can be managed by dose reduction as part of efficient combination therapies, the advantages of niclosamide and its salicylanilide analogs as antifungal drugs are considerable. Salicylanilides possess new mechanisms of action that are not covered by currently available antimycotic compound classes. Hence, they have the potential to prevent or overcome resistance to currently applied drugs. Since niclosamide was shown to possess distinct antiviral activities against SARS-CoV-2 and HIV, and fungal co-infections occur in several viral infections (e.g., cryptococcocal meningitis in HIV patients and candidiasis, aspergillosis, and mucormycosis in COVID-19 patients), antifungal salicylanilides can kill two birds with one stone and tackle both life-threatening infections in patients [132,133,134,135]. The pharmacological properties of niclosamide are well elaborated and provide useful hints at a proper administration of the drug for patients suffering from various mycoses. Several clinical studies of niclosamide (or its formulations) have been started for the therapy of various cancers, COVID-19 infection, diabetic nephropathy, rheumatoid arthritis, ulcerative colitis, and familial adenomatous polyposis [36]. The data generated from these trials can contribute to the accurate design of future clinical trials with salicylanilide drugs for the therapy of human mycoses. Availability of the drug is also achieved at relatively low costs. For instance, suitable niclosamide drugs for the therapy of COVID-19 and other respiratory diseases including fungal infections can be easily made by the extraction of Yomesan pills [136]. In addition to Bayer, there are more suppliers of generic niclosamide drugs from Asia (HAB Pharmaceuticals and Research Ltd. in India and Hanzhong Tianyuan Pharmaceuticals in China), and together with a simple preparation, its availability for a global and poor group of patients suffering from mycoses can be vouchsafed easily [136]. Moreover, small-molecule salicylanilides are usually very stable and can be stored and transported without problems, which is of importance for the management of mycoses, in particular, of fungal NTDs, in countries with under-developed health care systems and challenging climatic conditions.

## 6. Conclusions

Salicylanilides such as niclosamide have shown promising antifungal activities and can become new salient antifungal drugs for various problematic fungal infections. Their mechanisms of action add well to the currently available antimycotics and have the potential to prevent and overcome drug resistance. Drawbacks such as low solubility and bioavailability, severe toxicities, and adverse effects can be managed by chemical fine-tuning of the salicylanilide molecule, by sophisticated formulations, and by suitable combinations with other antifungals. The simple preparation of salicylanilides and the broad availability of niclosamide and other salicylanilide-based veterinary drugs are further hallmarks of this valuable compound class. More preclinical studies on their antifungal mechanisms and drug-like properties are necessary to enter clinical trials. But given the current clinical knowledge of niclosamide for the therapy of other human diseases, the development of a new antimycotic salicylanilide drug seems to be possible and desirable. 

## Data Availability

Not applicable.

## References

[1] Kainz K., Bauer M.A., Madeo F., Carmona-Gutierrez D. (2020). Fungal infections in humans: The silent crisis. Microb. Cell.

[2] Friedman D.Z.P., Schwartz I.S. (2019). Emerging fungal infections: New patients, new patterns, new pathogens. J. Fungi.

[3] Queiroz-Telles F., Fahal A.H., Falci D.R., Caceres D.H., Chiller T., Pasqualatto A.C. (2017). Neglected endemic mycoses. Lancet Infect. Dis..

[4] Arendrup M.C., Patterson T.F. (2017). Multidrug-resistant *Candida*: Epidemiology, molecular mechanisms, and treatment. J. Infect. Dis..

[5] Rodrigues M.L., Nosanchuk J.D. (2020). Fungal diseases as neglected pathogens: A wake-up call to public health officials. PLoS Negl. Trop. Dis..

[6] Mycetoma. https://dndi.org/diseases/mycetoma/.

[7] Araújo G.R.S., Souza W., Frases S. (2017). The hidden pathogenic potential of environmental fungi. Future Microbiol..

[8] Téllez M.D., Batista-Duharte A., Portuondo D., Quinello C., Bonne-Hernández R., Carlos I.Z. (2014). *Sporothrix schenkii* complex biology: Environment and fungal pathogenicity. Microbiology.

[9] Carmo A., Rocha M., Pereirinha P., Tomé R., Costa E. (2023). Antifungals: From pharmacokinetics to clinical practice. Antibiotics.

[10] Van Daele R., Spriet I., Wauters J., Maertens J., Mercier T., Van Hecke S., Brüggemann R. (2019). Antifungal drugs: What brings the future?. Med. Mycol..

[11] Gintjee T.J., Donnelley M.A., Thompson G.R. (2020). Aspiring antifungals: Review of current antifungal pipeline developments. J. Fungi.

[12] Jampilek J. (2022). Novel avenues for identification of new antifungal drugs and current challenges. Expert Opin. Drug Discov..

[13] 125 Years of Acetylsalicylic Acid. https://www.bayer.com/en/news-stories/125-years-of-acetylsalicylic-acid.

[14] Andrews P., Thyssen J., Lorke D. (1983). The biology and toxicology of molluscicides, Bayluscide. Pharmacol. Ther..

[15] Jara M.O., Williams III R.O. (2023). The challenge of repurposing niclosamide: Considering pharmacokinetic parameters, routes of administration, and drug metabolism. J. Drug Deliv. Sci. Technol..

[16] Molnar N.M. (1968). Influence of various surfactants on the antimicrobial activity of bromsalans and other ring-halogenated substances. J. Am. Oil Chem. Soc..

[17] Xia Q., Tan P., Feng X., Chen M., Kajihara N., Minai M., Hosaka Y. (1992). Assessment of the molluscicidal activities of tribromosalan, cartap and chlorothalonil against *Oncomelania hupensis*. Jpn. J. Med. Sci. Biol..

[18] Swan G.E. (1999). The pharmacology of halogenated salicylanilides and their anthelmintic use in animals. J. S. Afr. Vet. Assoc..

[19] Pearson I.G., Whitlock H.V., DeGoosh C.P., Farrington K.J., Jones R.C., Haigh J.A. (1970). Clioxanide, a new anthelmintic active against *Fasciola hepatica* and *Haeminchus contortus* in sheep. Aust. Vet. J..

[20] Kadri H., Lambourne O.A., Mehellou Y. (2018). Niclosamide, a drug with many (re)purposes. ChemMedChem.

[21] Chen W., Mook R.A., Premont R.T., Wang J. (2018). Niclosamide: Beyond an antihelminthic drug. Cell Signal..

[22] Kapale S.S., Chaudhari H.K. (2021). Niclosamide & challenges in chemical modifications: A broad review on enhancement of solubility. J. Indian Chem. Soc..

[23] Wang Z., Ren J., Du J., Wang H., Liu J., Wang G. (2022). Niclosamide as a promising therapeutic player in human cancer and other diseases. Int. J. Mol. Sci..

[24] Kauerová T., Pérez-Pérez M.-J., Kollar P. (2023). Salicylanilides and their anticancer properties. Int. J. Mol. Sci..

[25] Mahdi J.G., Mahdi A.J., Mahdi A.J., Bowen I.D. (2006). The historical analysis of aspirin discovery, its relation to the willow tree and antiproliferative and anticancer potential. Cell Prolif..

[26] Hirwe N.W., Rana K.N., Gavankar K.D. (1938). Derivatives of salicylic acid. Part XIII. Chlorosalicylic acids and their methyl ethers. Proc. Indian Acad. Sci. A.

[27] Chae H.-D., Cox N., Capolicchio S., Lee J.W., Horikoshi N., Kam S., Ng A.A., Edwards J., Butler T.-L., Chan J. (2019). SAR optimization studies on modified salicylamides as a potential treatment for acute myeloid leukemia through inhibition of the CREB pathway. Bioorg. Med. Chem. Lett..

[28] Juang Y.-P., Chou Y.-T., Lin R.-X., Ma H.-H., Chao T.-L., Jan J.-T., Chang S.-Y., Liang P.-H. (2022). Design, synthesis and biological evaluations of niclosamide analogues against SARS-CoV-2. Eur. J. Med. Chem..

[29] Lal J., Ramalingam K., Meena R., Ansari S.B., Saxena D., Chopra S., Goyal N., Reddy D.N. (2023). Design and synthesis of novel halogen rich salicylanilides as potential antileishmanial agents. Eur. J. Med. Chem..

[30] Li Y.-R., Lin C.-C., Huang C.-Y., Wong Y.-H., Hsieh C.-H., Wu H.-W., Chen J.J.W., Wu Y.-S. (2017). Study of the inhibitory effects on TNF-α-induced NF-κB activation of IMD0354 analogs. Chem. Biol. Drug Des..

[31] Niclosamide Oral. https://medicalguidelines.msf.org/en/viewport/EssDr/english/niclosamide-oral-16684320.html.

[32] Espinosa-Aguirre J.J., Reyes R.E., de Nava C.C. (1991). Mutagenic activity of 2-chloro-4-nitroaniline and 5-chlorosalicylic acid in *Salmonella typhimurium*: Two possible metabolites of niclosamide. Mutat. Res..

[33] Beristain-Castillo E., Martínez-Vázquez M., Camacho-Carranza R., Espinosa-Aguirre J.J. (2013). CYP1A1 and Cnr nitroreductase bio-activated niclosamide in vitro. Mutagenesis.

[34] Chang Y.-W., Yeh T.-K., Lin K.-T., Chen W.-C., Yao H.-T., Lan S.-J., Wu Y.-S., Hsieh H.-P., Chen C.-M., Chen C.-T. (2006). Pharmacokinetics of anti-SARS-CoV agent niclosamide and its analogs in rats. Yaowu Shipin Fenxi.

[35] Lu D., Ma Z., Zhang T., Zhang X., Wu B. (2016). Metabolism of the anthelmintic drug niclosamide by cytochrome P450 enzymes and UDP-glucuronosyltransferases: Metabolite elucidation and main contributions from CYP1A2 and UGT1A1. Xenobiotica.

[36] Seo J.I., Jin G.-W., Yoo H.H. (2024). Pharmacokinetic considerations for enhancing drug repurposing opportunities of anthelmintics: Niclosamide as a case study. Biomed. Pharmacother..

[37] Fan X., Li H., Ding X., Zhang Q.-Y. (2019). Contributions of hepatic and intestinal metabolism to the disposition of niclosamide, a repurposed drug with poor bioavailability. Drug Metab. Dispos..

[38] Mook Jr R.A., Wang J., Ren X.-R., Chen M., Spasojevic I., Barak L.S., Lyerly H.K., Chen W. (2015). Structure-activity studies of Wnt/β-catenin inhibition in the niclosamide chemotype: Identification of derivatives with improved drug exposure. Bioorg. Med. Chem..

[39] Ngai T.W., Elfar G.A., Yeo P., Phua N., Hor J.H., Chen S., Ho Y.S., Cheok C.F. (2021). Nitro-deficient niclosamide confers reduced genotoxicity and retains mitochondrial uncoupling activity for cancer therapy. Int. J. Mol. Sci..

[40] Chang X., Zhen X., Liu J., Ren X., Hu Z., Zhou Z., Zhu F., Ding K., Nie J. (2017). The anthelmintic phosphate niclosamide impedes renal fibrosis by inhibiting homeodomain-interacting protein kinase 2 expression. Kidney Int..

[41] Barbosa E.J., Löbenberg R., de Araujo G.L.B., Bou-Chacra N.A. (2019). Niclosamide repositioning for treating cancer: Challenges and nano-based drug delivery opportunities. Eur. J. Pharm. Biopharm..

[42] Choi G., Piao H., Rejinold N.S., Yu S., Kim K.-Y., Jin G.-W., Choy J.-H. (2021). Hydrotalcite–niclosamide nanohybrid as oral formulation towards SARS-CoV-2 viral infections. Pharmaceuticals.

[43] Jara M.O., Warnken Z.N., Sahakijpijarn S., Moon C., Maier E.Y., Christensen D.J., Koleng J.J., Peters J.I., Hackman Maier S.D., Williams R.O. (2021). Niclosamide inhalation powder made by thin-film freezing: Multi-dose tolerability and exposure in rats and pharmacokinetics in hamsters. Int. J. Pharm..

[44] Bruce J.I., Miller R., Lightner L., Yoganathan S. (1992). Efficacy of niclosamide as a potential topical antipenetrant (TAP) against cercariae of *Schistosoma mansoni* in monkeys. Mem. Inst. Oswaldo Cruz.

[45] Wulff C., Haeberlein S., Haas W. (2007). Cream formulations protecting against cercarial dermatitis by *Trichobilhazia*. Parasitol. Res..

[46] Weiss A., Delavenne E., Matias C., Lagler H., Simon D., Li P., Hansen J.U., dos Santos T.P., Jana B., Priemel P. (2022). Topical niclosamide (ATx201) reduces *Staphylococcus aureus* colonization in atopic dermatitis patients in a randomized, double-blind, placebo-controlled phase 2 trial. Clin. Transl. Med..

[47] Chung Y.-C., Kim G.Y., Kim D.E., Choi J.W., Chun B.C. (2019). Grafting of niclosamide and salicylanilide onto hydrophilic polyurethane for the control of fungal and barnacle growth. Polym. Bull..

[48] Kirk P.M., Cannon P.F., Minter D.W., Stalpers J.A. (2008). Dictionary of the Fungi.

[49] Brandt M.E., Lockhart S.R. (2012). Recent taxonomic developments with *Candida* and other opportunistic yeasts. Curr. Fungal Infect. Rep..

[50] Spettel K., Kriz R., Wu C., Achter L., Schmid S., Galazka S., Selitsch B., Camp I., Makristathis A., Lagler H. (2023). *Candida auris* in Austria—What is new and what is different. J. Fungi.

[51] Garcia C., Burgain A., Chaillot J., Pic É., Khemiri I., Sellam A. (2018). A phenotypic small-molecule screen identifies halogenated salicylanilides as inhibitors of fungal morphogenesis, biofilm formation and host cell invasion. Sci. Rep..

[52] Sutar Y., Nabeela S., Singh S., Alqarihi A., Solis N., Ghebremariam T., Filler S., Ibrahim A.S., Date A., Uppuluri P. (2022). Niclosamide-loaded nanoparticles disrupt *Candida* biofilms and protect mice from mucosal candidiasis. PLoS Biol..

[53] Mamouei Z., Singh S., Lemire B., Gu Y., Alqarihi A., Nabeela S., Li D., Ibrahim A., Uppuluri P. (2021). An evolutionary diverged mitochondrial protein controls biofilm growth and virulence in *Candida albicans*. PLoS Biol..

[54] Abdel-Rahman S.M., Simon S., Wright K.J., Ndjountche L., Gaedigk A. (2006). Tracking *Trichophyton tonsurans* through a large urban childcare center: Defining infection prevalence and transmission patterns by molecular stain typing. Pediatrics.

[55] Sidrim J.J.C., Rocha M.F.G., Leite J.J.G., da Albuquerque Maranhao F.C., Lima R.A.C., Castelo-Branco D.S.C.M., Bandeira T.J.P.G., Cordeiro R.A., Brilhante R.S.N. (2013). *Trichophyton tonsurans* strains from Brazil: Phenotypic heterogeneity, genetic homology, and detection of virulence genes. Can. J. Microbiol..

[56] Abdel-Rahman S.M., Wright K.J., Navarre H. (2009). Griseofulvin has only a modest impact on eradicating carriage of *Trichophyton tonsurans*. J. Pediatr. Pharmacol. Ther..

[57] Preuett B., Leeder J.S., Abdel-Rahman S. (2015). Development and application of a high-throughput screening method to evaluate antifungal activity against *Trichophyton tonsurans*. J. Biomol. Screen..

[58] Waller S.B., Lana D.F.D., Quatrin P.M., Ferreira M.R.A., Fuentefria A.M., Mezzari A. (2021). Antifungal resistance on *Sporothrix* species: An overview. Braz. J. Microbiol..

[59] Xavier M.O., Poester V.R., Trápaga M.R., Stevens D.A. (2023). *Sporothrix brasiliensis*: Epidemiology, therapy, and recent developments. J. Fungi.

[60] Almeida-Paes R., de Andrade I.B., Ramos M.L.M., de Araújo Rodrigues M.V., do Nascimento V.A., Bernardes-Engemann A.R., Frases S. (2021). Medicines for Malaria Venture COVID Box: A source for repurposing drugs with antifungal activity against human pathogenic fungi. Mem. Inst. Oswaldo Cruz.

[61] Ramos M.L.M., Almeida-Silva F., de Souza Rabello V.B., Nahal J., Figueiredo-Carvalho M.H.G., Bernardes-Engemann A.R., Poester V.R., Xavier M.O., Meyer W., Zancopé-Oliveira R.M. (2024). In vitro activity of the anthelmintic drug niclosamide against *Sporothrix* spp. strains with distinct genetic and antifungal susceptibility backgrounds. Braz. J. Microbiol..

[62] Van de Sande W.W.J. (2013). Global burden of human mycetoma: A systematic review and meta-analysis. PLoS Negl. Trop. Dis..

[63] Elkheir L.Y.M., Haroun R., Mohamed M.A., Fahal A.H. (2020). *Madurella mycetomatis* causing eumycetoma medical treatment: The challenges and prospects. PLoS Negl. Trop. Dis..

[64] World’s First Clinical Trial for Devastating Fungal Disease Mycetoma Shows Efficacy of New, Promising Treatment. https://dndi.org/press-releases/2023/worlds-first-clinical-trial-for-mycetoma-shows-efficacy-new-promising-treatment/.

[65] Ma J., Todd M., van de Sande W.W.J., Biersack B. (2023). Antifungal activity of natural naphthoquinones and anthraquinones against *Madurella mycetomatis*. Chem. Biodivers..

[66] Mahmoud A.B., Abd Algaffar S., van de Sande W., Khalid S., Kaiser M., Mäser P. (2021). Niclosamide is active in vitro against mycetoma pathogens. Molecules.

[67] Hibbett D.S., Blackwell M., James T.Y., Spatafora J.W., Taylor J.W., Vilgalys R. (2018). Phylogenetic taxon definitions for Fungi, Dikarya, Ascomycota and Basidiomycota. IMA Fungi.

[68] Park B.J., Wannemuehler K.A., Marston B.J., Govender N., Pappas P.G., Chiller T.M. (2009). Estimation of the current global burden of cryptococcal meningitis among persons living with HIV/AIDS. Aids.

[69] Bahn Y.-S., Sun S., Heitman J., Lin X. (2020). Microbe profile: *Cryptococcus neoformans* species complex. Microbiology.

[70] Bicanic T., Meintjes G., Wood R., Hayes M., Rebe K., Bekker L.G., Harrison T. (2007). Fungaly burden, early fungicidal activity, and outcome in cryptococcal meningitis in antiretroviral-naïve and antiretroviral-experienced patients treated with amphotericin B or fluconazole. Clin. Infect. Dis..

[71] Dehdashti S.J., Abbott J., Nguyen D.-T., McKew J.C., Williamson P.R., Zheng W. (2013). A high throughput screening assay for assessing viability of *Cryptococcus neoformans* under nutrient starvation condition. Anal. Bioanal. Chem..

[72] Ortiz S.C., Huang M., Hull C.M. (2019). Spore germination as a target for antifungal therapeutics. Antimicrob. Agents Chemother..

[73] Beck D.P., Greenawalt J.W. (1976). Biogenesis of mitochondrial membranes in *Neurospora crassa* during cellular differentiation: Changes in oxidative phosphorylation and synthesis of mitochondrial phospholipids. J. Gen. Microbiol..

[74] Terenzi H.F., Storck R. (1969). Stimulation of fermentation and yeast-like morphogenesis in *Mucor rouxii* by phenethyl alcohol. J. Bacteriol..

[75] Broome A.W., Jones G.M. (1966). A new drug for the treatment of fascioliasis in sheep and cattle. Nature.

[76] OpenSourceMycetoma. https://github.com/OpenSourceMycetoma.

[77] Pic E., Burgain A., Sellam A. (2019). Repurposing the anthelmintic salicylanilide oxyclozanide against susceptible and clinical resistant *Candida albicans* strains. Med. Mycol..

[78] Peyclit L., Yousfi H., Rolain J.-M., Bittar F. (2021). Drug repurposing in medical mycology: Identification of compounds as potential antifungals to overcome the emergence of multidrug-resistant fungi. Pharmaceuticals.

[79] Zhang J., Bai Y., Li B., Zhou X., Si H., Zhang J. (2019). Determination and pharmacokinetics study of oxyclozanide suspension in cattle by LC-MS/MS. BMC Vet. Res..

[80] Wang W., Dong Z., Zhang J., Zhou X., Wei X., Cheng F., Li B., Zhang J. (2019). Acute and subacute toxicity assessment of oxyclozanide in Wistar rats. Front. Vet. Sci..

[81] Vieira J.N., Maia Filho F.S., Ferreira G.F., Mendes J.F., Goncalves C.L., Villela M.M., Pereira D.I.B., Nascente P.S. (2017). In vitro susceptibility of nematophageous fungi to antiparasitic drugs: Interactions and implications for biological control. Braz. J. Biol..

[82] Michiels M., Meuldermans W., Heykants J. (1987). The metabolism and fate of closantel (Flukiver) in sheep and cattle. Drug Metab. Rev..

[83] Swan G.E., Koeleman H.A., Steyn H.S., Mülders M.S. (1999). Intravascular plasma disposition and salivary secretion of closantel and rafoxanide in sheep. J. S. Afr. Vet. Assoc..

[84] Leary E.O., Gasior S., McElnea E. (2023). Closantel toxicity. BMJ Case Rep..

[85] Bazvand F., Riazi-Esfahani H., Salari F. (2023). Presumed veterinary niclosamide-induced retinal toxicity in a human: A case report. J. Med. Case Rep..

[86] Swan G.E., Schröder J. (1981). A safety trial with rafoxanide in sheep. J. S. Afr. Vet. Assoc..

[87] Schröder J. (1982). The safety of injectable rafoxanide in cattle. J. S. Afr. Vet. Assoc..

[88] Bendary M.M., Abd El-Hamid M.I., Abousaty A.I., Elmanakhly A.R., Alshareef W.A., Mosbah R.A., Alhomrani M., Ghoneim M.M., Elkelish A., Hashim N. (2023). Therapeutic switching of rafoxanide: A new approach to fighting drug-resistant bacteria and fungi. Microbiol. Spectr..

[89] Norbury H.M., Waring R.H. (1981). The disposition and metabolism of 3,4′,5-tribromosalicylanilide and 4′,5-dibromosalicylanilide in the rat. Xenobiotica.

[90] Daidone G., Raffa D., Plescia S., Matera M., Caruso A., Leone V., Amico-Roxas M. (1989). Synthesis and evaluation of the analgesic and antiinflammatory activities of *N*-substituted salicylamides. Farmaco.

[91] Marhold J. (1986). Prehled Prumyslove Toxicologie: Organicke Latky.

[92] Mitchell S.C., Norbury H.M., Waring R.H., Gadsden P.M., Wood P.B. (1982). A comparison of the metabolism and elimination of benzanilide and salicylanilide in the rat. Xenobiotica.

[93] Krátký M., Vinšova J. (2011). Salicylanilide ester prodrugs as potential antimicrobial agents–a review. Curr. Pharm. Des..

[94] Krátky M., Vinšova J., Buchta V., Horvati K., Bösze S., Stolaříková J. (2010). New amino acid esters of salicylanilides active against MDR-TB and other microbes. Eur. J. Med. Chem..

[95] Krátký M., Vinšová J., Buchta V. (2012). In vitro antibacterial and antifungal activity of salicylanilide benzoates. Sci. World. J..

[96] Krátký M., Vinšová J. (2012). Antifungal activity of salicylanilides and their esters with 4-(trifluoromethyl)benzoic acid. Molecules.

[97] Krátký M., Vinšová J., Buchta V. (2012). In vitro antibacterial and antifungal activity of salicylanilide pyrazine-2-carboxylates. Med. Chem..

[98] Krátký M., Vinšová J. (2016). Salicylanilide *N*-monosubstituted carbamates: Synthesis and in vitro antimicrobial activity. Bioorg. Med. Chem..

[99] Appetecchia F., Consalvi S., Scarpecci C., Biava M., Poce G. (2020). SAR analysis of small molecules interfering with energy-metabolism in *Mycobacterium tuberculosis*. Pharmaceuticals.

[100] Paraskevopoulos G., Monteiro S., Vosátka R., Krátký M., Navrátilová L., Treitnar F., Stolaříková J., Vinšová J. (2017). Novel salicylanilides from 4,5-diahalogenated salicylic acids: Synthesis, antimicrobial activity and cytotoxicity. Bioorg. Med. Chem..

[101] Ng M.Y., Song Z.J., Tan C.H., Bassetto M., Hagen T. Structural investigations on the mitochondrial uncouplers niclosamide and FCCP. FEBS Open Bio.

[102] Prichard R.K. (1978). The metabolic profile of adult *Fasciola hepatica* obtained from rafoxanide-treated sheep. Parasitology.

[103] Hossain S., Veri A.O., Liu Z., Iyer K.R., O’Meara T.R., Robbins N., Cowen L.E. (2021). Mitochondrial perturbation reduces susceptibility to xenobiotics through altered efflux in *Candida albicans*. Genetics.

[104] Cheng S., Clancy C.J., Nguyen K.T., Clapp W., Nguyen M.H. (2007). A *Candida albicans* petite mutant strain with uncoupled oxidative phosphorylation overexpresses MDR1 and has diminished susceptibility to fluconazole and voriconazole. Antimicrob. Agents Chemother..

[105] Liston S.D., Whitesell L., Kapoor M., Shaw K.J., Cowen L.E. (2020). Enhanced efflux pump expression in *Candida* mutants results in decreased manogepix susceptibility. Antimicrob. Agents Chemother..

[106] Koltai T. (2022). The complex relationship between multiple drug resistance and the tumor pH gradient: A review. Cancer Drug Resist..

[107] Lionakis M.S. (2023). Exploiting antifungal immunity in the clinical context. Semin. Immunol..

[108] Al-Gareeb A.I.A., Gorial F.I., Mahmood A.S. (2018). Niclosamide as an adjuvant to etanercept in treatment patients with active rheumatoid arthritis: An 8-week randomized controlled pilot study. Clin. Rheumatol..

[109] Krátký M., Dzurková M., Janoušek J., Konecná K., Trejtnar F., Stolaříková J., Vinšová J. (2017). Sulfadiazine salicylaldehyde-based Schiff bases: Synthesis, antimicrobial activity and cytotoxicity. Molecules.

[110] Bonilla J.J.A., Honorato L., Haranahalli K., Gremiao I.D.F., Pereira S.A., Guimaraes A., de Souza Baptista A.R., de Melo Tavares P., Rodrigues M.L., Miranda K. (2021). Antifungal activity of acylhydrazone derivatives against *Sporothrix* spp.. Antimicrob. Agents Chemother..

[111] Ferreira E.S., Cordeiro L.V., Silva D.F., Souza H.D.S., de Athayde-Filho P.F., Barbosa-Filho J.M., Scotti L., Lima E.O., de Castro R.D. (2021). Antifungal activity and mechanism of action of 2-chloro-*N*-phenylacetamide: A new molecule with activity against strains of *Aspergillus flavus*. An. Acad. Bras. Cienc..

[112] Diniz-Neto H., Silva S.L., Cordeiro L.V., Silva D.F., Oliveira R.F., Athayde-Filho P.F., Oliveira-Filho A.A., Guerra F.Q.S., Lima E.O. (2024). Antifungal activity of 3-chloro-*N*-phenylacetamide: A new molecule with fungicidal and antibiofilm activity against fluconazole-resistant *Candida* spp.. Braz. J. Biol..

[113] Silva S.L., de Oliveira Pereira F., Cordeiro L.V., Diniz-Neto H., dos Santos Maia M., da Silva Souza H.D., de Athayde-Filho P.F., Scotti M.T., Scotti L., de Oliveira Lima E. (2022). Antifungal activity of 2-chloro-*N*-phenylacetamide, docking and molecular dynamics studies against clinical isolates of *Candida tropicalis* and *Candida parapsilosis*. J. Appl. Microbiol..

[114] Dos Santos Ferreira E., Cordeiro L.V., de Figueredo Silva D., Diniz-Neto H., de Sousa A.P., da Silva Souza H.D., de Athayde-Filho P.F., Guerra F.Q.S., Barbosa-Filho J.M., de Oliveira Filho A.A. (2024). Evaluatuon of antifungal activity, mechanisms of action and toxicological profile of the synthetic amide 2-chloro-*N*-phenylacetamide. Drug Chem. Toxicol..

[115] Copp J.N., Pletzer D., Brown A.S., van der Heijden J., Miton C.M., Edgar R.J., Rich M.H., Little R.F., Williams E.M., Hancock R.E.W. (2020). Mechanistic understanding enables the rational design of salicylanilide combination therapies for Gram-negative infections. mBio.

[116] Lateef M., Zargar S.A., Khan A.R., Nazir M., Shoukat A. (2008). Successful treatment of niclosamide- and praziquantel-resistant beef tapeworm infection with nitazoxanide. Int. J. Infect. Dis..

[117] Song H.-N., Jeong D.-G., Bang S.-Y., Paek S.-H., Park B.-C., Park S.-G., Woo E.-J. (2015). Crystal structure of the fungal nitroreducates Frm2 from *Saccharomyces cerevisiae*. Protein Sci..

[118] Zhou Y., Lv H., Li H., Li J., Yan Y., Liu F., Hao W., Zhou Z., Wang P., Zhou S. (2021). Nitroreductase increases menadione-mediated oxidative stress in *Aspergillus nidulans*. Appl. Environ. Microbiol..

[119] Wojcicki A., Chae H.-D., Han K., Youn M., Wilkes M.C., Lacayo N.J., Bassik M., Sakamoto K.M. (2020). Genetic modulators of niclosamide sensitivity and resistance in acute myeloid leukemia. Blood.

[120] Stover K.R., Hawkins B.K., Keck J.M., Barber K.E., Cretella D.A. (2023). Antifungal resistance, combinations and pipeline: Oh my!. Drugs Context.

[121] Wiederhold N.P. (2020). Review of the novel investigational antifungal olorofim. J. Fungi.

[122] Fajardo-Orduña G.R., Ledesma-Martínez E., Aguiñiga-Sánchez I., Mora-García M.d.L., Weiss-Steider B., Santiago-Osorio E. (2021). Inhibitors of chemoresistance pathways in combination with ara-C to overcome multidrug resistance in AML. A mini review. Int. J. Mol. Sci..

[123] Guo Y., Zhu H., Xiao Y., Guo H., Lin M., Yuan Z., Yang X., Huang Y., Zhang Q., Bai Y. (2022). The anthelmintic drug niclosamide induces GSK-β-mediated β-catenin degradation to potentiate gemcitabine activity, reduce immune evasion ability and suppress pancreatic cancer progression. Cell Death Dis..

[124] Vermes A., Guchelaar H.-J., Dankert J. (2000). Flucytosine: A review of its pharmacology, clinical indications, pharmacokinetics, toxicity and drug interactions. J. Antimicrob. Chemother..

[125] Nitzsche B., Höpfner M., Biersack B. (2023). Synthetic small molecule modulators of Hsp70 and Hsp40 chaperones as promising anticancer agents. Int. J. Mol. Sci..

[126] Rouges C., Asad M., Laurent A.D., Marchand P., Le Pape P. (2023). Is the C-terminal domain an effective and selective target for the design of Hsp90 inhibitors against *Candida* yeast?. Microorganisms.

[127] Behboudi H., Alizadeh Y., Medghalchi A., Soltani-Moghadam R., Azaripour E., Moravvej Z. (2023). Closantel retinal toxicity: Recovery from severe vision loss after corticosteroid therapy. Eur. J. Ophthalmol..

[128] Kang M.-J., Kim M.J., Kim A., Koo T.-S., Lee K.-R., Chae Y.-J. (2024). Pharmacokinetic interactions of niclosamide in rats: Involvement of organic anion transporters 1 and 3 and organic cation transporter 2. Chem. Biol. Interact..

[129] Vermeer L.M.M., Isringhausen C.D., Ogilvie B.W., Buckley D.B. (2016). Evaluation of ketoconazole and its alternative clinical CYP3A4/5 inhibitors as inhibitors of drug transporters: The in vitro effects of ketoconazole on 13 clinically-relevant drug transporters. Drug Metab. Dispos..

[130] Xu S., Feliu M., Lord A.K., Lukason D.P., Negoro P.E., Khan N.S., Dagher Z., Feldman M.B., Reedy J.L., Steiger S.N. (2018). Biguanides enhance antifungal activity against *Candida glabrata*. Virulence.

[131] Kang H.E., Seo Y., Yun J.S., Song S.H., Han D., Cho E.S., Cho S.B., Jeon Y., Lee H., Kim H.S. (2021). Metformin and niclosamide synergistically suppress Wnt and YAP in APC-mutated colorectal cancer. Cancers.

[132] Singh S., Weiss A., Goodman J., Fisk M., Kulkarni S., Lu I., Gray J., Smith R., Sommer M., Cheriyan J. (2022). Niclosamide–a promising treatment for COVID-19. Br. J. Pharmacol..

[133] Niyomdecha N., Suptawiwat O., Boonarkart C., Jitobaom K., Auewarakul P. (2020). Inhibition of human immunodeficiency virus type 1 by niclosamide through mTORC1 inhibition. Heliyon.

[134] Sharma A., Bano G., Malik A., Rasool Y., Manzar S., Singh T., Maity M. (2023). Opportunistic fungal invasion in COVID-19 pandemic: A critical review in diagnosis and management. Avicenna J. Med..

[135] Qureshi Z.A., Ghazanfar H., Altaf F., Ghazanfar A., Hasan K.Z., Kandhi S., Fortuzi K., Dileep A., Shrivistava S. (2024). Cryptococcosis and cryptococcal meningitis: A narrative review and the up-to-date management approach. Cureus.

[136] Needham D. (2023). Extraction of niclosamide from commercial approved tablets into aqueous buffered solution creates potentially approvable oral and nasal sprays against COVID-19 and other respiratory infections. AAPS Open.

